# Characterization of Five Novel *Brevibacillus* Bacteriophages and Genomic Comparison of *Brevibacillus* Phages

**DOI:** 10.1371/journal.pone.0156838

**Published:** 2016-06-15

**Authors:** Jordan A. Berg, Bryan D. Merrill, Justin T. Crockett, Kyle P. Esplin, Marlee R. Evans, Karli E. Heaton, Jared A. Hilton, Jonathan R. Hyde, Morgan S. McBride, Jordan T. Schouten, Austin R. Simister, Trever L. Thurgood, Andrew T. Ward, Donald P. Breakwell, Sandra Hope, Julianne H. Grose

**Affiliations:** Department of Microbiology and Molecular Biology, Brigham Young University, Provo, UT, United States of America; ContraFect Corporation, UNITED STATES

## Abstract

*Brevibacillus laterosporus* is a spore-forming bacterium that causes a secondary infection in beehives following European Foulbrood disease. To better understand the contributions of *Brevibacillus* bacteriophages to the evolution of their hosts, five novel phages (Jenst, Osiris, Powder, SecTim467, and Sundance) were isolated and characterized. When compared with the five *Brevibacillus* phages currently in NCBI, these phages were assigned to clusters based on whole genome and proteome synteny. Powder and Osiris, both myoviruses, were assigned to the previously described Jimmer-like cluster. SecTim467 and Jenst, both siphoviruses, formed a novel phage cluster. Sundance, a siphovirus, was assigned as a singleton phage along with the previously isolated singleton, Emery. In addition to characterizing the basic relationships between these phages, several genomic features were observed. A motif repeated throughout phages Jenst and SecTim467 was frequently upstream of genes predicted to function in DNA replication, nucleotide metabolism, and transcription, suggesting transcriptional co-regulation. In addition, paralogous gene pairs that encode a putative transcriptional regulator were identified in four *Brevibacillus* phages. These paralogs likely evolved to bind different DNA sequences due to variation at amino acid residues predicted to bind specific nucleotides. Finally, a putative transposable element was identified in SecTim467 and Sundance that carries genes homologous to those found in *Brevibacillus* chromosomes. Remnants of this transposable element were also identified in phage Jenst. These discoveries provide a greater understanding of the diversity of phages, their behavior, and their evolutionary relationships to one another and to their host. In addition, they provide a foundation with which further *Brevibacillus* phages can be compared.

## Introduction

Due to their ability to transfer genetic material or to lyse and kill their hosts, the study of bacteriophages is critical to understanding the evolution of their host bacteria [1]. This evolution-driving genetic exchange is known as horizontal gene transfer (HGT) and can also operate conversely where phages acquire genetic fragments from their bacterial host [2]. Phages are also capable of sharing genes with other closely or distantly-related phages through HGT, leading to “genetic shuffling” of phage genetic components [3]. These gene transfer events frequently occur through recombination pathways or the use of transposable elements marked by inverted repeats in the DNA [4]. An additional source of evolutionary change in phages is through single-nucleotide mutagenesis events leading to differing protein activities, such as differential binding patterns of transcription factors or altered enzymatic activity. Thus, it is no surprise that the fast evolutionary rate of phages, propelled in part by HGT, as well as the sheer abundance of phages, has been shown to play a vital role in the evolution of many pathogenic bacterial strains [5], and is likely a driving force in the evolution of *Brevibacillus laterosporus*.

*Brevibacillus laterosporus* (BL) is one of several secondary invaders often associated with *Melissococcus plutonius* infection, the causative agent of European foulbrood in honeybees [6]. Like *Paenibacillus larvae* (PL), the causative agent of American Foulbrood, *B*. *laterosporus* belongs to the bacterial family *Paenibacillaceae* [7], forms endospores, and is commonly found in beehives [8]. While *P*. *larvae* has only been found in beehives, *B*. *laterosporus* has been isolated from the gut of healthy adult honeybees and is found in a multitude of other sources [8,9]. Understanding the diversity within natural isolates of various beehive bacteria and their interplay with one another, as well as with their respective phages, can be key in understanding beehive health (including whether the concerted infection of both *B*. *laterosporus* and *P*. *larvae* or *M*. *plutonius* has a symbiotic relationship) and may enhance the efforts to prevent further devastation from beehive disease.

In addition to causing secondary infection in honeybees, *B*. *laterosporus* has several properties that are potentially useful in biocontrol applications. First, it has shown strong insecticidal activity via gut liquefaction [10,11] and is toxic to mosquitoes that carry dangerous human diseases [12,13]. Because it is also active against many fly larvae, *B*. *laterosporus* can be added to animal feed to control fly populations that reproduce in animal feces [11]. However, no insecticidal properties have been reported in honeybees [9,14]. Second, it has broad-spectrum antimicrobial activity [8] through peptides that inhibit growth of Gram-positive and Gram-negative bacteria, as well as fungi [15], and these peptides are resistant to heat, proteases, and pH changes. Third, *B*. *laterosporus* can metabolize many waste compounds and may be useful in bioremediation [8]. Understanding the nature and evolution of *B*. *laterosporus* and its phages is, therefore, advantageous in an effort to harness these potential biocontrol properties of this bacterium.

Five *Brevibacillus* phages were previously isolated and characterized [16]. All five were myoviruses and were grouped into one cluster (containing phages Jimmer1, Jimmer2, Abouo, and Davies) and one singleton (Emery) based upon whole genome and proteome analysis. Herein we report the isolation and characterization of five novel *B*. *laterosporus* phages: Powder, Osiris, Jenst, SecTim467, and Sundance. Jenst, SecTim467, and Sundance are the first siphoviruses to be isolated that infect *Brevibacillus* bacteria. These new phages add an additional cluster and an additional singleton, expanding previously limited *Brevibacillus* phage relationships to a current total of two clusters and two singletons based on genome synteny. Genomic comparisons of the ten *Brevibacillus* phages reveals interesting motif features likely involved in transcriptional control, conserved and repeated proteins of interest, and a putative transposable region present in several of these phages. In addition, several proteins were identified that could contribute to the pathogenicity of *Brevibacillus*, including a bacterial pili regulatory protein; a PAAR repeat protein, which may aid in secretion and killing of target cells [17]; both the stage V sporulation proteins K and T, which allow for normal sporulation of the host to occur; and many others.

## Materials and Methods

### *Brevibacillus* isolation, culture and plating

*B*. *laterosporus* field isolates BL2 or BL6, previously described as PL2 and PL6 [16,18], or BL14 were used for phage isolation in this study. BL14 was isolated in the same manner as previous field isolates, but from a diseased larva displaying classical symptoms of American Foulbrood. Briefly, samples of honey, beehive material, and bee larvae were processed for intended *P*. *larvae* isolation by isolating spores [19] and incubating on PLA plates at 37°C [20]. Gram-positive isolates that were catalase-negative [21] were further streaked and purified on LB (Lennox) agar. Broth cultures of all isolates that were used for plaque assays and host range studies were inoculated in LB Lennox broth and incubated while shaking (200 rpm) at 37°C overnight. Bacterial lawns were plated by mixing 500 μL of overnight culture with 4.5 mL of 0.8% LB top agar.

### Phage infection of *Brevibacillus* bacteria

*Brevibacillus* bacteria were tested for phage susceptibility by using a plaque assay as well as a spot test assay. For the plaque assay, phage lysate was incubated at room temperature with 500 μL of an overnight culture of bacteria for 30 minutes, plated in 0.8% LB top agar, and incubated overnight at 37°C. For the spot test assay, 500 μL of an overnight culture of bacteria was plated in 0.8% top agar. After the top agar hardened, 3 μL of phage lysate was placed on the top agar. The plates were incubated overnight at 37°C with the agar side facing up.

### Isolation of *Brevibacillus* phages

*Brevibacillus* phages were isolated from bee debris collected near beehives. Bee debris was crushed using a micro pestle and added to a flask containing LB broth along with a culture of *B*. *laterosporus* field isolates BL2, BL6, or BL14. The bee debris and bacteria were incubated overnight at 37°C. The mixture was spun in a centrifuge (8,000 rpm for 10 minutes) and the supernatant was passed through a 0.22 μm Nalgene SFCA filter (Thermo Scientific). Depending on initial phage titer, 5–50 μL of the supernatant was incubated at room temperature with 500 μL of BL bacteria for 30–60 minutes, mixed with LB top agar, plated on LB agar, and incubated at 37°C overnight. Plaques were sampled using a sterile pipette tip and the process of plaque purification was repeated at least three times to isolate each phage.

### Transmission Electron microscopy

Samples were prepared for transmission electron microscopy as previously described [16] and imaged at the BYU Microscopy Center.

### DNA extraction, sequencing, assembly, and annotation

Phage DNA was extracted using the Phage DNA Isolation Kit (Norgen Biotek Corporation). DNA was extracted according to the manufacturer’s instructions, except 5–10 times the lysate amount was used with the appropriate reagents scaled up proportionally. Phage DNA was sequenced using 454 sequencing technology and sequencing reads were assembled using Newbler 2.9 (454 Life Sciences, Roche), Consed [22], and Gepard [23]. Phage genomes were screened for direct terminal repeats using PAUSE (https://cpt.tamu.edu/computer-resources/pause) and were annotated using DNA Master [24] and other programs described previously [18,25].

### Identification of phage host range

The spot test assay described above was used to verify host susceptibility to each of the phages. *Brevibacillus* phage lysates obtained as described above were used to infect field isolates BL2, BL6, BL14, and two additional *B*. *laterosporus* strains (40A1 (KF597228) [26] and 40A9 (KF597235) [27]) obtained from the Bacillus Genetic Stock Center (www.bgsc.org), or BGSC.

### Phylogenomic analysis of phage genes and genomes

Dotplots were prepared using Gepard [23] and phage clusters were defined based on approximately 50% or greater genome synteny as previously described [3,28,29]. Phamerator [30] was used to analyze and prepare full-genome comparison maps using a representative member of each cluster. Full-genome accession numbers used in preparing dotplots and in conducting other genomic analyses for this study are:

*Brevibacillus* phages Abouo (*B*. *laterosporus*) [KC595517], Davies (*B*. *laterosporus*) [NC_022980], Emery (*B*. *laterosporus*) [KC595516], Jenst (*B*. *laterosporus*) [KT151955], Jimmer1 (*B*. *laterosporus*) [KC595515], Jimmer2 (*B*. *laterosporus*) [KC595514], Osiris (*B*. *laterosporus*) [KT151956], Powder (*B*. *laterosporus*) [KT151958], SecTim467 (*B*. *laterosporus*) [KT151957], Sundance (*B*. *laterosporus*) [KT151959].

Kalign [31–35] was used to calculate average nucleotide identity (ANI) of phage genomes. Proteomic analysis of each of the phage clusters was performed using CoreGenes [36–38] with the BLASTP score threshold set at 75.

Sequences of closest phage relatives for phylogenetic comparisons were found from BLASTP [39–41] searches using the amino acid sequence of each of the phages’ terminase large subunit or terminase large subunit-like protein that was at least 25% identical to the target *Brevibacillus* phage terminase large subunit or terminase large subunit-like protein. Sequences for phylogenetic analysis were first aligned using MUSCLE [42]. Neighbor-joining phylogenetic trees were prepared by MEGA6 [43] with bootstrapping set to 500. Any branches with a bootstrapping value less than 50% were collapsed. No out-group was included as phage terminases are highly susceptible to horizontal gene transfer [44]. The accession numbers for the terminase large subunit or terminase large subunit-like proteins used in this study are:

*Bacillus* phage SPP1 (*B*. *subtilis*) [NP_690654]; *Brevibacillus* phages Abouo (*B*. *laterosporus*) [AGR47449], Davies (*B*. *laterosporus*) [YP_008858637], Emery (*B*. *laterosporus*) [AGR47349], Jenst (*B*. *laterosporus*) [ALA07151], Jimmer1 (*B*. *laterosporus*) [AGR47249], Jimmer2 (*B*. *laterosporus*) [AGR47149], Osiris (*B*. *laterosporus*) [ALA07344], Powder (*B*. *laterosporus*) [ALA48012], SecTim467 (*B*. *laterosporus*) [ALA07521], Sundance (*B*. *laterosporus*) [ALA47835]; *Clostridium* phages c-st (*C*. *botulinum*) [YP_398598], D-1873 (*C*. *botulinum*) [EES90358]; *Lactococcus* phages phiL47 (*L*. *lactis* subsp. *cremoris* DPC6860) [YP_009007060], 949 (*L*. *lactis* strain ML8) [YP_004306307]; *Microcystis* phages Ma-LMM01 (*M*. *aeruginosa*) [YP_851132], MaMV-DC (*M*. *aeruginosa*) [AGR48682]; *Pseudomonas* phage PaBG (*P*. *aeruginosa* PAO1) [YP_008433440]; *Staphylococcus* phages PH15 (*S*. *epidermidis*) [YP_950664]; *Streptomyces* phage Jay2Jay (*S*. *lividans* JI1326) [AIW02598]; *Synechoccus* phage S-CBS4 (*Synechococcus* sp. CB0101) [YP_005098309]; *Thermus* phages phiOH2 (*T*. *thermophilus*) [YP_008240332], phiYS40 (*T*. *thermophilus*) [YP_874075], TMA (*T*. *thermophilus* HB27) [YP_004782268]; *Treponema* bacterial strain *Treponema phagedenis* F0421 [EFW38857].

### Motif Identification and Analysis

MEME [45] was used to scan phage genomes for statistically significant motifs (P-value < 1e-3, Q-value < 0.05). FIMO [46] was used to scan the phage and bacterial genomes and determine the presence and exact positions of each iteration of the motif. DNA Master [24] and Phamerator [30] were then used to determine the genes neighboring discovered motifs in each of the phage genomes.

### Identification of conserved domains

Conserved domains for all *Brevibacillus* phages were found using the NCBI Conserved Domain Database [47–50] with the acceptable return threshold set at E-value < 1e-3.

### Structural prediction of proteins encoded by paralogous genes

Phamerator was used to group similar proteins into protein families called “phams” [30] using parameters identical to those used by Merrill et al. [16]. Protein families containing multiple homologs from the same phage were then identified. Clustal Omega [51] was used to compare the amino acid sequences of the paralogous proteins. CD-Search [47–50] was used to find proteins with similar conserved domains. *Brevibacillus* phage conserved domains were compared to the c2 repressor of *Salmonella* phage P22 (*S*. *enterica* serovar typhimurium) [1ADR_A, 2R1J_L, 3JXB_C] [52]. HHPred [53] and RaptorX [54–57] were used to predict tertiary structure and binding sites. These predicted structures were then superimposed using STRAP [58] and compared using NCBI’s Cn3D [59].

### Transposon Region

Phamerator [30] was used to analyze the differentiating features between Jenst and SecTim467. The Palindrome command of EMBOSS [60] was used to verify the presence of inverted repeats surrounding the transposable region. A dotplot comparing the homologous region in *Brevibacillus laterosporus* LMG 15441 [NZ_CP007806] to the putative transposon region in SecTim467 was prepared using Gepard [23]. Using the default parameters of HHPred [53], homologous proteins within the putative transposable region were discovered. Protein folding predictions were then accessed from the RCSB Protein Data Bank [61] based on hits from HHPred using an acceptable threshold of E-value < 1e-12. GC content of the putative transposon region located between the inverted repeats was calculated using the online GC content calculator at endmemo.com (http://www.endmemo.com/bio/gc.php).

## Results and Discussion

### Five Novel *Brevibacillus* phages belong to the families Myoviridae and Siphoviridae

In total, five new *Brevibacillus* phages were recently isolated from bee debris or bee larvae collected along northern Utah using field isolates BL2, BL6, and BL14 (Table 1) and these phages were later sequenced. Phages Powder and Sundance were co-isolated and not separated prior to electron microscopy or sequencing. These genomes assembled completely as two separate contigs. Following isolation, and in order to confirm their identity as *B*. *laterosporus* phages, these phages were used to infect two well-characterized *B*. *laterosporus* strains from BGSC (*B*. *laterosporus* strains 40A1 and 40A9). All of the five novel *Brevibacillus* phages isolated were capable of infecting the BGSC strains used, namely *B*. *laterosporus* 40A1 and 40A9, confirming that these phages infect hosts belonging to the *Brevibacillus* genus. Along with being able to infect these BGSC strains and their isolation strain (indicated by an underlined + in Table 1), Powder and Sundance were also able to infect BL2, SecTim467 was able to infect BL6, and Osiris and SecTim467 were able to infect BL14.

**Table 1:** *Brevibacillus* phages isolated in this study.

Phage Name	Sample location	Sampletype	GenBank Accession	Fold coverage	Genome length (bp)	ORFs	tRNAs	GC (%)	Host 40A1	Host 40A9	Host BL2	Host BL6	Host BL14
Powder	Eden, UT	Bee debris	KT151958	73.528	52,992	103	0	38.1	+	+	+	-	+
Osiris	Salt Lake City, UT	Bee larva	KT151956	351.6	52,955	103	0	38.1	+	+	+	-	+
Jenst	Salt Lake City, UT	Bee debris	KT151955	87.3	126,341	178	6	42.9	+	+	-	+	-
SecTim467	Orem, UT	Bee debris	KT151957	122.9	130,482	183	6	42.7	+	+	+	+	+
Sundance	Eden, UT	Bee debris	KT151959	20.425	134,270	194	0	35.5	+	+	+	-	+

“Fold coverage” is the average depth of sequencing coverage for each of these phages. “ORFs” is the number of open reading frames annotated. Isolation host is underlined and describes the strain used to initially isolate the phage, while all listed phages contained in the table additionally infected both of the BGSC strains used in this study. + indicates that the phage listed was capable of infecting the given strain for host range studies, whereas–indicates an inability of the phage being able to infect the given strain. An underlined + indicates the original isolation host.

**Fig 1:**
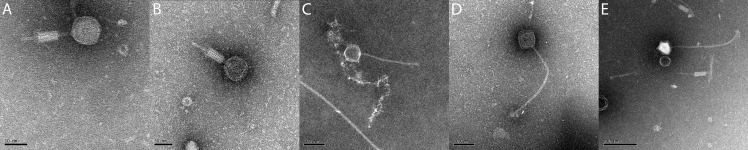
Transmission electron microscopy of five novel *B*. *laterosporus* phages identifies two myoviruses and three siphoviruses. (A) *Brevibacillus* phage Powder. (B) *Brevibacillus* phage Osiris. (C) *Brevibacillus* phage Jenst. (D) *Brevibacillus* phage SecTim467. (E) *Brevibacillus* phage Sundance. Samples were prepared for transmission electron microscopy as previously described by Merrill et al. [16].

Transmission electron micrographs of the five new phages indicate they are either myoviruses or siphoviruses (Fig 1). The structure of Powder and Osiris suggest they are myoviruses, as were all five of the previously characterized *Brevibacillus* phages [16]. Jenst, SecTim467, and Sundance are the first siphoviruses to be reported that infect *Brevibacillus* bacteria. Measurements of the capsid and tail structures of these phages are listed in Table 2. As expected, phages of the same cluster share the same morphology as all members of the Jimmer-like cluster are myoviruses [16] and all members of the Jenst-like cluster are siphoviruses (updated and new *Brevibacillus* phage clusters are discussed below). Note that there are myovirus tail particles in the Sundance image due to co-isolation with Powder.

**Table 2:** Structural measurements of *Brevibacillus* phages from transmission electron micrograph images.

Phage Name	Capsid height (nm)	Capsid width (nm)	Tail length (nm)	Tail width (nm)	Contracted sheath length (nm)	Contracted sheath width (nm)	Morphology
Powder	65.7 ± 1.7	63.9 ± 5.3	120.3 ± 7.9	7.9 ± 1.2	52 ± 11.2	23.9 ± 5.0	Myovirus
Osiris	64.0 ± 4.0	63.7 ± 3.7	114.8 ± 5.8	7.2 ± 0.8	49.2 ± 3.7	20.9 ± 1.4	Myovirus
Jenst	86.8 ± 6.1	86.9 ± 8.3	286.2 ± 22.5	11.7 ± 2.0	N/A	N/A	Siphovirus
SecTim467	96.8 ± 5.8	87.9 ± 5.8	414.6 ± 23.9	13.6 ± 2.5	N/A	N/A	Siphovirus
Sundance	96.4 ± 4.0	94.4 ± 4.7	403.7 ± 36.8	13.0 ± 1.97	N/A	N/A	Siphovirus

Measurements reported are average lengths of three or more instances of the structures in nanometers with standard deviations as determined in ImageJ [62]. Jenst, SecTim467, and Sundance are siphoviruses and do not possess sheaths, thus no measurements were available for these structural characteristics and these cells are marked “N/A”.

### Phylogenomics of *Brevibacillus* phages

**Fig 2:**
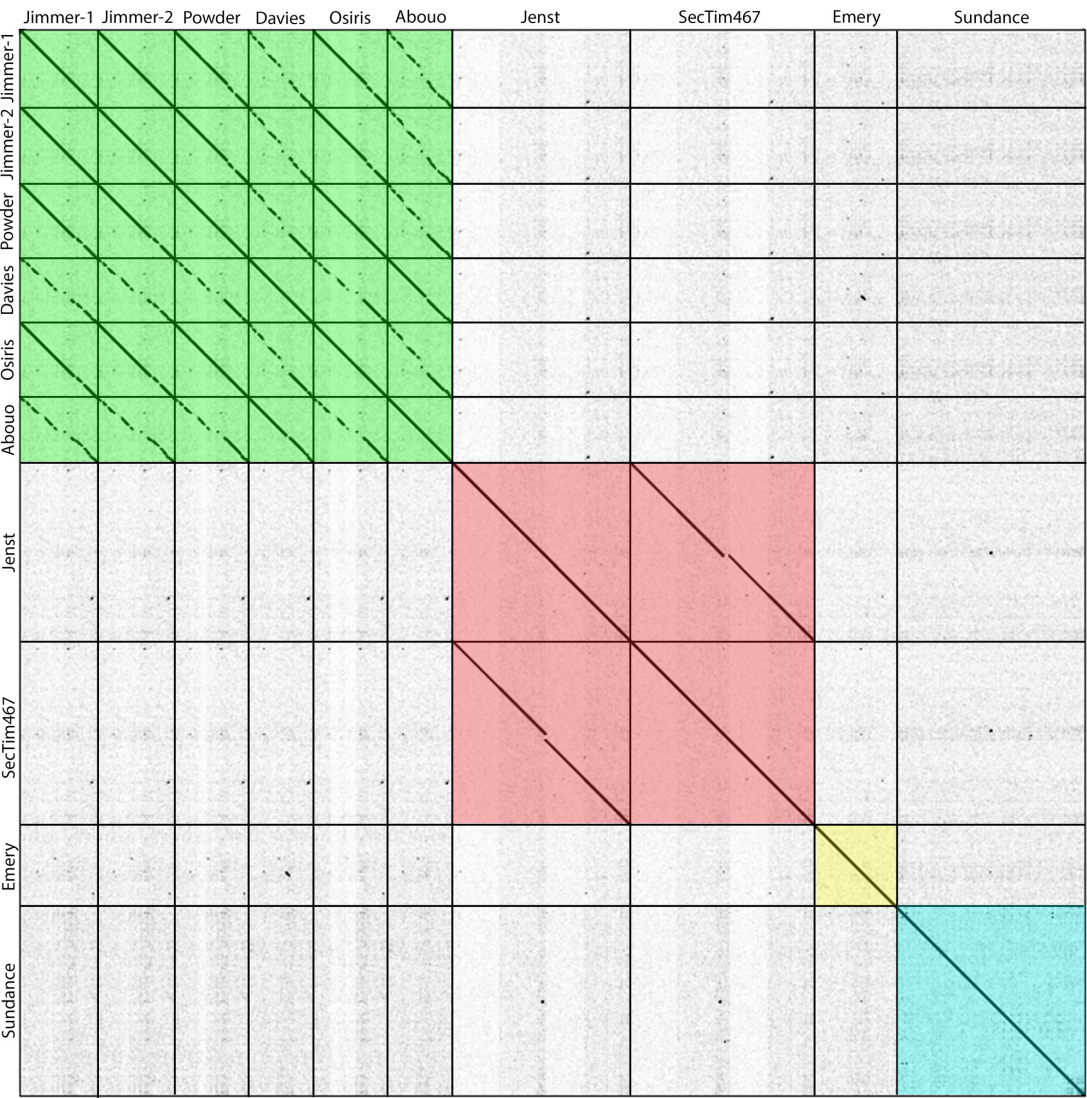
Whole-genome nucleotide dotplot analysis of *Brevibacillus* phages reveals four distinct clusters. Gepard [23] nucleotide dotplot reveals four distinct phage clusters: the Jimmer-like cluster, the Jenst-like cluster, and the Emery and Sundance singletons. Diagonal lines indicate genome similarity. Vertical and horizontal lines were added to indicate phage genome boundaries and clusters of related phages are highlighted in different colors. Single dots present are a result of smaller DNA fragments aligning with that of other phages and do not represent sufficient similarity to elicit placing a phage into a particular cluster. Analysis window with the word size of 10 base pairs was used.

**Fig 3:**
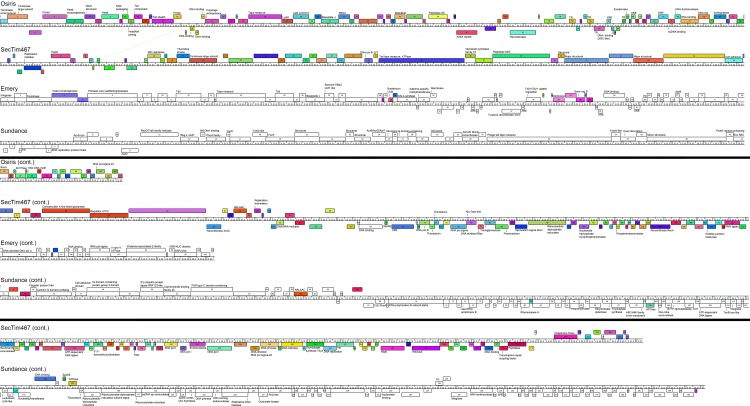
Whole-genome comparison maps show diversity between *Brevibacillus* phage clusters. A Phamerator [30] whole genome comparison map was prepared using a representative member of each cluster. Boxes on top of the genome ruler indicate genes that are expressed in the forward direction while those below the ruler are expressed in the reverse direction. Gene products are numbered. Colored boxes correspond to encoded proteins that belong to a particular pham (family of homologous proteins) found in any of the ten *Brevibacillus* phages, while white boxes denote an orpham (an ORF not belonging to a pham). Purple, red, or green lines between genomes illustrate regions of nucleotide similarity. Emery gps 4–5 represents a frameshift event by ribosomal slippage. Functions annotated were compiled from both BLAST and CDD searches. Abbreviations used include: MGEBNA (mannosyl-glycoprotein endo-beta-N-acetylglucosamidase); XRE (XRE family transcriptional regulator); TR (Trascriptional regulator); ssDNA binding (single-stranded DNA binding); DNA rep (DNA replication); RecU (recombinase RecU); SSp DNA meth (Site-specific DNA methylase); RNA pol sigma 24 (RNA polymerase sigma 24); MD hydrolase (Metal-dependent hydrolase); DNA pol III G/T (DNA polymerase III subunit gamma/tau); Regulator of CC (Regulator of chromosome condensation family); NALAAC (N-acetylmuramoyl-L-alanine amidase CwlA); Spo0E (Sporulation protein Spo0E OR Spo0E-like sporulation regulator family); DNA pol III (DNA polymerase III); RNA pol sigma (RNA polymerase sigma factor); FtsK (Cell division protein FtsK); DNA pol I (DNA polymerase I); NALA amidase (N-acetylmuramoyl-L-alanine amidase); XRE (XRE family transcriptional regulator); Gene reg B (Accessory gene regulator B family); DNA-directed DNA pol A (DNA-directed DNA polymerase, family A); RNA pol sigma (RNA polymerase sigma factor); V spor K, ATPase (stage V sporulation protein K, ATPase AAA); Ac-di syn (cobyrinic acid ac-diamide synthase); XerD (site specific recombinase XerD); Reg s, luxR (bacterial regulatory s, luxR family); BN DNA binding (Bacterial nucleoid DNA-binding); Exc ABC (Excinuclease ABC); CJE RusA (Crossover junction endodeoxyribonuclease RusA); Asp-tRNA amidotrans B (Aspartyl-tRNA amidotransferase subunit B); ssDNA-sp exonuclease (Single-stranded DNA-specific exonuclease).

In order to determine the relationships of these five novel phages to the pre-existing field of *Brevibacillus* phages, all ten fully sequenced *Brevibacillus* phages were genomically compared. Phages are often grouped into clusters based on genome synteny over at least 50% of the phage genome [3,28,29]. Based on >50% genome synteny, as seen by whole-genome dotplot analysis (Fig 2) and confirmed by Kalign [31–35] average nucleotide identity (ANI) analysis, *Brevibacillus* phages were grouped into two clusters and two singletons. Osiris and Powder belonged to the previously defined Jimmer-like cluster including Jimmer1, Jimmer2, Davies, and Abouo, while a novel Jenst-like cluster emerged including Jenst and SecTim467. Sundance and Emery were designated as singletons. It should be noted that ANI values within these assigned clusters is >75% and intercluster ANI is <55% indicating broad nucleotide relationships among the ten *B*. *laterosporus* phages and their respective clusters (Table 3). Fig 3 provides a genomic map of a representative member from each cluster, as well as both singletons, produced using Phamerator [30]. These maps reveal extremely low or no nucleotide (marked by the lack of purple, red, or green lines between genomes) and protein (marked by the lack of similarly colored boxes) similarity between the phage clusters. These relationships allude to either the divergent evolution of very distinct clusters of *Brevibacillus* phages, as has been reported for the *Mycobacteriophages*, *Enterobacterioaceae* phages, and *Bacillus* phages [3,28,63], or that there are a multitude of unknown *Brevibacillus* phages that will merge these gaps in the future. It should also be noted that Kalign ANI values of Jenst and SecTim467 showed them to be ~100% identical, but SecTim467 is 4,141 bps longer than Jenst. The entire genome of Jenst is 100% similar to the equivalent sequence in SecTim467. A predicted transposable region found in SecTim467 explains the difference of 4,141 total bps and break in sequence similarity in SecTim467 as compared to Jenst (visible on the dotplot in Fig 2) and is discussed further below.

**Table 3:** Average Nucleotide Identity of *Brevibacillus laterosporus* phages suggests four clusters of related phages.

Phage Name	Jimmer1	Jimmer2	Powder	Davies	Osiris	Abouo	Jenst	SecTim467	Emery	Sundance
Jimmer1	100%									
Jimmer2	99.83%	100%								
Powder	96.36%	96.53%	100%							
Davies	77.50%	77.57%	82.05%	100%						
Osiris	95.59%	95.76%	99.26%	81.81%	100%					
Abouo	76.26%	76.33%	78.89%	93.35%	78.63%	100%				
Jenst	42.92%	42.96%	43.14%	40.96%	43.24%	40.44%	**100%**	** **		
SecTim467	43.02%	43.06%	43.23%	41.04%	43.32%	40.54%	**100%**	**100%**		
Emery	45.47%	45.49%	45.64%	44.47%	45.58%	41.53%	39.77%	39.64%	*100%*	
Sundance	52.07%	52.12%	53.28%	47.62%	53.59%	46.56%	43.96%	44.03%	39.26%	*100%*

Average nucleotide identity was determined by Kalign [31–35]. Clusters of related phages are noted with bold, italics, and underlining. Jenst and SecTim467 were reported as having 100% nucleotide identity by Kalign, however SecTim467 contains an additional 4,141 bps. This table is reporting the 126,341 bps that are analogous between these two phages. Values underlined or bolded denote comparisons being made within the same cluster. Singletons are italicized.

These cluster assignments agree with whole proteome analysis of these phages using CoreGenes [36–38]. Coregenes analysis revealed high levels of proteome conservation within the Jimmer-like cluster, as well as between Jenst and SecTim467 (Table 4), further confirming the clusters previously determined by nucleotide synteny. Related phages are reported to have at least 40% of their proteomes conserved [64,65], making the CoreGenes-determined proteomic clusters defined by these values consistent with the cluster assignments previously proposed in this study where intercluster values are greater than 65% and intracluster are less than 13%.

**Table 4:** Whole proteomic analysis supports *Brevibacillus* phage cluster assignments.

Phage Name	Jimmer1	Jimmer2	Powder	Davies	Osiris	Abouo	Jenst	SecTim467	Emery	Sundance
Jimmer1	103 (100%)									
Jimmer2	103 (100%)	103 (100%)								
Powder	97 (94.17%)	97 (94.17%)	103 (100%)							
Davies	65 (69.15%)	65 (69.15%)	69 (73.4%)	94 (100%)						
Osiris	96 (93.2%)	96 (93.2%)	102 (99.03%)	69 (66.99%)	103 (100%)					
Abouo	62 (65.96%)	62 (65.96%)	64 (68.09%)	85 (90.43%)	64 (68.09%)	94 (100%)				
Jenst	7 (3.93%)	7 (3.93%)	6 (3.37%)	4 (2.25%)	6 (3.37%)	5 (2.81%)	**178 (100%)**			
SecTim467	7 (3.93%)	7 (3.93%)	6 (3.28%)	4 (2.19%)	6 (3.28%)	6 (3.28%)	**178 (97.3%)**	**183 (100%)**		
Emery	10 (9.8%)	10 (9.8%)	8 (7.84%)	11 (10.78%)	8 (7.84%)	9 (8.82%)	7 (6.86%)	8 (7.84%)	*102 (100%)*	
Sundance	4 (2.06%)	4 (2.06%)	4 (2.06%)	5 (2.58%)	4 (2.06%)	4 (2.06%)	23 (11.86%)	25 (12.89%)	4 (2.06%)	*194 (100%)*

Proteomic synteny as determined using Coregenes [36–38]. Clusters of related phages are noted with bold, italics, and underlining. Results are based on a BLASTP score threshold of 75. Coregenes values of >40% have been previously used to identify related phages [64,65]. Values underlined or bolded denote comparisons being made within the same cluster. Singletons are italicized.

**Fig 4:**
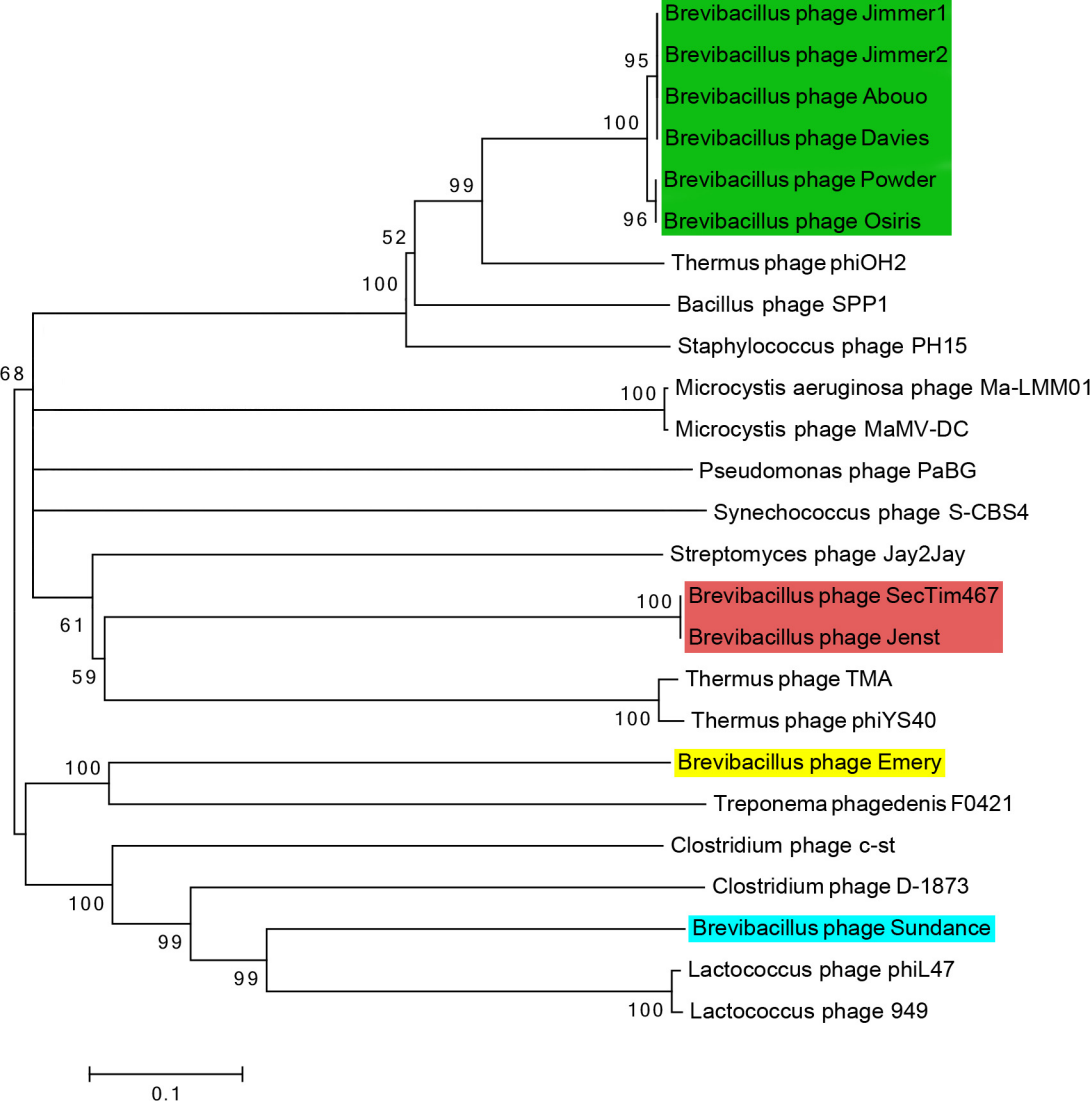
Terminase phylogenetic analysis supports four clusters of *Brevibacillus* phages with close relationships to phages from other hosts. Terminase phylogeny for *Brevibacillus* phages alongside other similar phage terminases based on at least 25% amino acid sequence shared identity. No outgroup was used as phage terminases are highly susceptible to horizontal gene transfer. Scale represents 0.1 amino acid substitutions per site. The phylogenetic tree was aligned using MUSCLE [67] and prepared using neighbor-joining trees in MEGA6 [43] with bootstrapping set to 500. Any branches with a bootstrapping value less than 50% were collapsed. Clusters of related phages are highlighted in different colors: the Jimmer-like cluster in green, the Jenst-like cluster in red, and the Emery and Sundance singletons in yellow and blue, respectively.

As phylogenetic analyses of the terminase large subunit protein sequences of phages have been used to group phages [66], analysis of the terminases of the *Brevibacillus* phages was used to further confirm whole genome cluster assignments in this study (Fig 4). By this terminase analysis, the Jimmer-like cluster phages were most similar to one another, and more similar to *Staphylococcus* phage PH15, *Bacillus* phage SPP1, and *Thermus* phage phiOH2 than to other *Brevibacillus phages*. Similarly, phages Jenst and SecTim467 were more closely related to one another and to *Streptomyces* phage Jay2Jay, as well as *Thermus* phages TMA and phiYS40, than to other *Brevibacillus* phages. The terminases from singleton *Brevibacillus* phages Emery and Sundance were also more closely related to other phages from other hosts than to *Brevibacillus* phages. Emery’s terminase is more closely related to a terminase found in the bacterium *Treponema phagedenis*, while the Sundance terminase is more closely related to *Lactococcus* phages phiL47 and phage 949 as well as *Clostridium* phages c-st and D-1873. While confirming the *Brevibacillus* phage cluster relationships presented in this paper, the terminase phylogenetic trees of these phages exhibit genetic shuffling as is often observed in phage genetics, as the representative phages for each of the phage clusters defined in this paper are more closely related to an unrelated phage than to any of the other *Brevibacillus* phage clusters.

### Evidence suggesting that eight of the ten *Brevibacillus* phages may be temperate phages

Determining whether a phage is temperate or lytic is often complicated by sub-optimal laboratory conditions for formation of lysogens or by difficulty identifying an integrase in the phage genome. Genomic searches for homology between the putative major capsid proteins of phages and genes within bacterial genomes can be used as a guide to determine whether a phage is temperate or lytic, as long as the reservoir of published sequences from the bacterial host is sufficiently large enough. BLASTP [39–41] analysis was therefore used to provide evidence of the lifestyle of the ten *Brevibacillus* phages. All of the phages in the Jimmer-like cluster (Jimmer1 [YP_009226318.1], Jimmer2 [AGR47154], Powder [ALA48018.1], Davies [YP_008858642.1], Osiris [YP_009215022] and Abouo [YP_009220064.1] were used as probes) have MCP BLASTP matches of 96% or greater similarity with E-values of 0 to proteins encoded by a *B*. *laterosporus* chromosome. In support of these phages being temperate, phages from the Jimmer-like cluster were previously shown to form stable lysogens [16]. While the remaining four phages did not have any experimentally confirmed MCPs and all putative MCPs (such as Jenst gp 26 and SecTim467 gp26) did not have high (~75% or greater identity) BLASTP matches to MCPs in bacterial genomes, both Emery and Sundance appeared to encode putative integrases, suggesting they may be temperate. The Emery integrase [AGR47348.1] call was based on a BLASTP match to an integrase encoded in a *P*. *elgii* chromosome with 69% similarity, E-value 9e-100, and the Sundance integrase [YP_009194207.1] call was based on a match to an integrase encoded in a *P*. *elgii* chromosome with 75% similarity, E-value 3e-123. However, no stable lysogens were formed from a lysate containing Emery [16]. Thus, based on BLASTP MCP analysis, as well as the presence of a putative integrase gene in some of the phage genomes, at least eight of the ten *Brevibacillus* phages (the Jimmer-like cluster phages as well as Emery and Sundance) may be temperate. Further work is necessary to test a variety of infection conditions that may facilitate lysogen formation and verify their lifestyles.

### Motif analysis reveals putative transcriptional units

**Fig 5:**
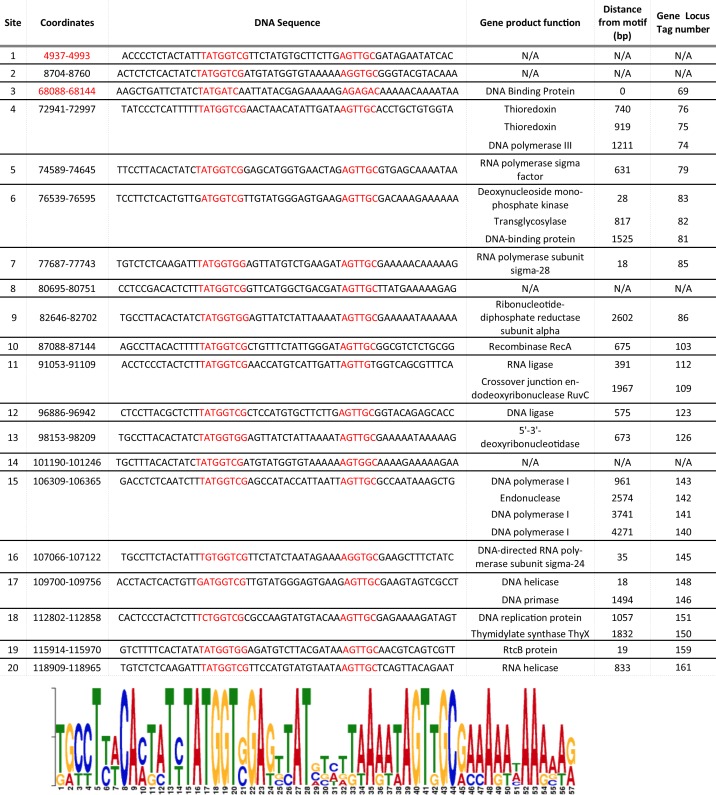
*Brevibacillus* phage SecTim467 genes harboring upstream conserved motifs are primarily involved in nucleotide metabolism/replication. FIMO [46] was used to locate the conserved motif in the genome of SecTim467. This chart also shows that these motifs are located in inter-gene gaps upstream of DNA metabolism and RNA transcription genes. “N/A” indicates the gene downstream of the motif at that location had an unknown function. The motif is displayed at the bottom, with the bases of the highly conserved portions of the motif highlighted throughout the figure in red. In many cases, several genes appear immediately downstream of the conserved motif in what may be an operon. In these cases, more than one gene function is reported. Two of the discovered motif sequences are present in the reverse complement of the given coordinates and are denoted by their coordinate numbers colored in red. The word graph at the bottom of the figure is a proportional representation of instances of different nucleotides at each position in the motif that contribute to the overall consensus sequence.

Motif analysis has been used to identify temporally co-transcribed units in phage genomes [68,69], leading to a greater understanding of the phage lifestyle. In an effort to predict transcriptional control methods of these novel phages, MEME [45] searches were performed and revealed a significant motif conserved in both SecTim467 and Jenst, but not found in Osiris, Powder, and Sundance. FIMO [46] identified this motif 20 times in SecTim467 (Fig 5) and 19 times in Jenst. BLASTN [39–41] searches were performed to determine if this motif was present in other phages and identified its prevalence in the recently characterized and otherwise unrelated *Bacillus* phage Basilisk [5]. By identifying the genes neighboring this motif in SecTim467, we hypothesized that the motif may act as a transcriptional regulator binding site as it is commonly located upstream of genes and the distance between the highly conserved portions of the motif structurally resembles a promoter binding site. In support of the use of this motif in transcriptional regulation, the genes downstream of this motif predominantly encode putative transcription and replication machinery (Fig 5), suggesting they may be co-transcribed for efficient replication of the phage genome. *Brevibacillus laterosporus* LMG 15441 was scanned for instances of this discovered motif without result, suggesting the phages might encode their own sigma factor or transcriptional regulator. In support of this idea, several putative genes in both Jenst and SecTim467 are homologous to sigma factors (Jenst: gp74 [YP_009199135.1], gp80 [YP_009199141.1], gp140 [YP_009199201.1]; SecTim467: gp79 [ALA07424.1], gp85 [ALA07429.1], gp145 [ALA07470.1]). It is therefore likely that this phage motif may be used to recruit sigma factors involved in transcribing middle or late expression phage genes as has been observed in *B*. *subtilis* phage SPO1 [70–72]. However, further study of this motif is required to determine whether or not it is in fact a promoter site, or whether it is acting in another manner.

### Conserved proteins, including putative virulence factors, conserved in *Brevibacillus* phages

In order to assess putative functions for the *Brevibacillus* phage proteins, as well as a possible role for these proteins in the evolution of these phages and their host, all conserved domains present within the ten *Brevibacillus* phage genomes were analyzed using Phamerator [30] and the NCBI Conserved Domain Database [47–50] (S1 Table). Several conserved domains corresponding to host functions as well as possible pathogenicity factors were present in these phages, suggesting that they may be contributing to the pathogenicity of their host. These include: the bacterial pili regulatory protein (Jimmer1 gp65 [YP_009226375.1], Jimmer2 gp65 [AGR47199.1]), which aids host attachment to its target cell [73]; the plasmid segregation protein ParM (Jenst gp6 [YP_009199067.1], SecTim467 gp6 [ALA07508.1]) and the plasmid partition protein ParA (Sundance gp7 [YP_009194057.1]), which allow for proper allocation of plasmid in dividing bacteria [74–76]; a PAAR repeat protein (Sundance gp40 [YP_009194090.1]), which may aid in secretion and killing of target cells [17]; both of the stage V sporulation proteins K (Emery gp95 [AGR47420.1]) and T (Emery gp72 [AGR47438.2]), which allow for normal sporulation of the host to occur [77,78]; along with several others (Table 5). The possession of these virulence factors by these phages may indicate the spread of pathogenicity genes from host to host with the phage acting as a vector via HGT between host and phage and/or vice versa [2], or that these genes may represent a “Trojan Horse” mechanism for host entry where the host recognizes the foreign DNA as advantageous and begins replication of the viral DNA.

**Table 5:** Conserved Domain search identifies several host virulence factors present in *Brevibacillus* phages.

				Gene product of the following phage:						
Putative Function	Jimmer1	Jimmer2	Powder	Davies	Osiris	Abouo	Jenst	SecTim467	Emery	Sundance
Sialic acid mutarotase	gp45	gp45	gp47		gp47					
Bacterial pili protein	gp65	gp65								
Plasmid segregation protein ParM							gp6	gp6		
Plasmid partition protein ParA										gp7
PhoH										gp15
PAAR repeat										gp40
Cell wall hydrolase										gp132
Stage V sporulation protein K									gp95	
Stage V sporulation protein T									gp72	

### Multiple copies of a helix-turn-helix DNA binding protein are found in four *Brevibacillus* phages

**Fig 6:**
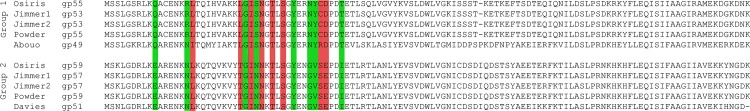
Multiple sequence alignment of Osiris gp55, Osiris gp59, and related homologs from *B*. *laterosporus* phages indicates high conservation. Two groups (Osiris gp55 and Osiris gp59), based on homology of related proteins, can be seen. The *Brevibacillus* phage name is followed by the gene product number and then the amino acid sequence of that gene product. Group numbers are provided to the left of the phage names. RaptorX [54–57] predictions for DNA binding residues are colored. Green residues differ in a group-specific manner while red residues are conserved between both groups.

**Fig 7:**
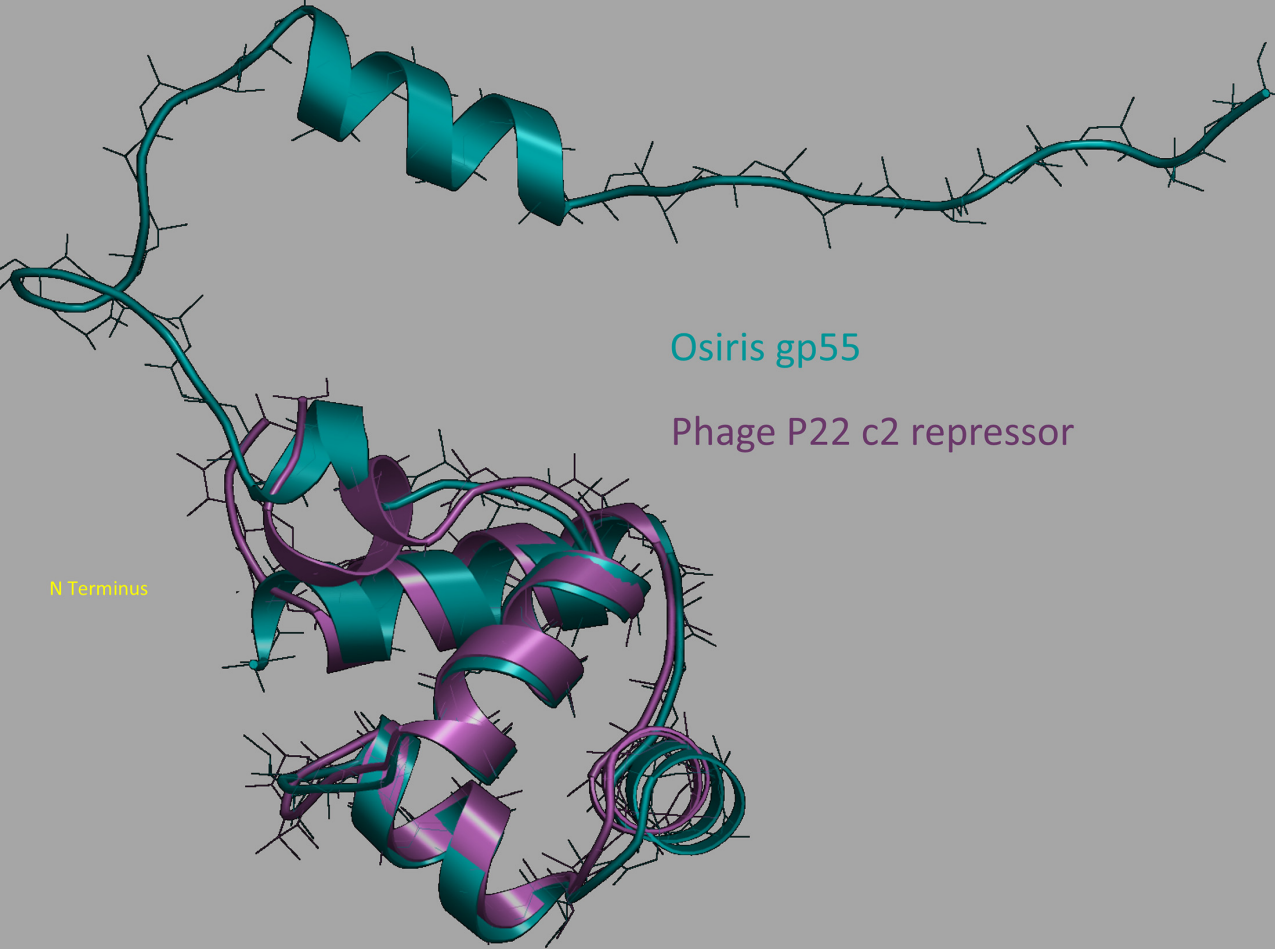
Osiris gp55 superimposed on the phage P22 c2 repressor shows similar N-terminal structure and suggests possible homodimerization prior to interaction with DNA. The predicted RaptorX [54–57] structure for Osiris gp55 superimposed using STRAP [58] on the phage P22 c2 repressor protein published by Watkins et al. [52,85] reveals high structural homology. The DNA binding domain as predicted by RaptorX [54–57] in Osiris gp55 is near the N-terminus, similar to the c2 repressor in phage P22. RaptorX was unable to accurately predict tertiary structure near the gp55 C-terminus.

Repeated genes often offer an evolutionary edge to their host, through either increasing the protein expression or the ability to evolve related but novel functions [79–82]. The *Brevibacillus* phages were analyzed for multiple gene copies within a single genome. Of particular interest from the list of conserved domains were two homologous putative helix-turn-helix transcriptional regulators found in phages of the Jimmer cluster (Jimmer1, Jimmer2, Osiris, and Powder). Abouo and Davies contained only one copy of the gene coding for this protein. These proteins formed two groups based on 60% or greater amino acid similarity. Group 1 consisted of Jimmer1 gp53 [YP_009226363.1], Jimmer2 gp53 [AGR47190.2], Abouo gp49 [YP_009220106.1], Osiris gp55 [YP_009215069.1], and Powder gp55 [ALA48065.1], while Group 2 consisted of Jimmer1 gp57 [YP_009226367.1], Jimmer2 gp57 [AGR47193.1], Davies gp51 [YP_008858686.1], Osiris gp59 [YP_009215073.1], and Powder gp59 [ALA48069.1]. Based on RaptorX [54–57] protein folding predictions, all protein sequences folded very similarly and predicted nucleic acid binding residues of these proteins varied in a group-specific manner (Fig 6). The variation of the nucleic acid pocket (or binding site) interactions between groups suggested that these transcription factors have evolved to bind different sequences for the two different groups, although Abouo and Davies differed somewhat from the group consensus (Table 6). This conserved protein showed some structural similarity (24–28% based on Clustal Omega alignment [51]) to the c2 repressor of *Salmonella* phage P22 [52,83–85] where the N-terminus of both structures showed consistent alignment (Fig 7). However, RaptorX was unable to predict tertiary structure of the C-terminus for any of the proteins within this protein family.

**Table 6:** DNA binding pocket comparison of Osiris gp55, Osiris gp59, and related homologous proteins from *B*. *laterosporus* phages.

Phage Name	Pocket 1	Pocket 2	Pocket 3	Pocket 4
**Group 1 Consensus**	**T**	**T**	**A**	**T**
Osiris gp55	T	T	A	T
Jimmer1 gp53	T	T	A	T
Jimmer2 gp53	T	T	A	T
Powder gp55	T	T	A	T
Abouo gp49	A	T	T	T
**Group 2 Consensus**	**T**	**T**	**T**	**T**
Osiris gp59	T	T	T	T
Jimmer1 gp57	T	T	T	T
Jimmer2 gp57	T	T	T	T
Powder gp59	T	T	T	T
Davies gp51	T	T	T	A

Display of the predicted nucleotide each pocket will bind to reveals overall conservation between each group with the exception of pocket 3, which shows independent evolution as Group 1 is predicted to bind adenine (except in Abouo gp49) while Group 2 is predicted to bind thymidine. Pocket binding predictions were performed by RaptorX [54–57]

Protein binding pockets, binding residues, or ligand-binding sites are cavities created by a protein’s secondary structure conformation. These pockets differentially bind different ligands, such as DNA, based on their conformation. The binding specificities of these sites can change based on single-base mutations in the gene coding for a given protein. By comparing these two groups of highly related proteins within the phages and the divergence of particular binding pockets, these distinct, yet related, phages appear to have independently evolved. While these paralogous genes most likely bind to slightly different DNA sequences, similarities between phage P22’s c2 repressor and the proteins in this protein family indicate that they still likely function similarly. As has been postulated in models for novel protein evolution, a duplicated gene would be able to accumulate random mutations, and if this series of mutations were to provide a novel, advantageous function, it could then be favored by selection [80]. Such proteins are referred to as Ohnologous proteins [86]. It is possible then that these phages are effectually beginning to modify and test these genes, providing a glimpse through the window of evolution.

### Transposable Region

**Fig 8:**
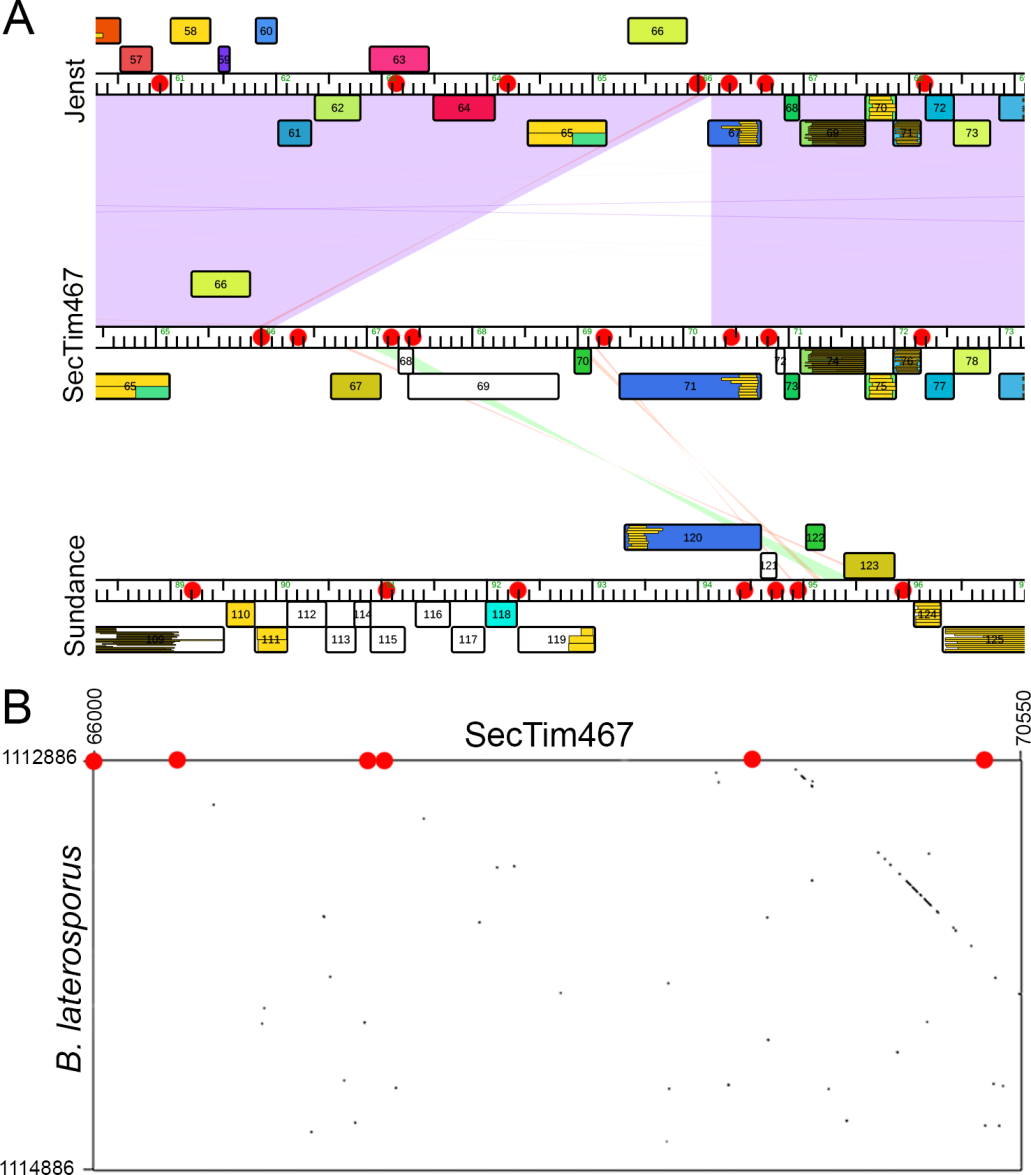
A Putative transposable region found in *Brevibacillus* phages shares homology with *B*. *laterosporus*. (A) Phamerator [30] genome map comparing transposable regions of Jenst (top), SecTim467 (middle), and Sundance (bottom). Colored boxes correspond to genes that belong to a particular pham (family of homologous proteins), while white boxes denote an orpham (an ORF not belonging to a pham). Purple, red, or green shading illustrates regions of nucleotide similarity. Boxes on top of the genome ruler indicate genes that are expressed in the forward direction while those below the ruler are expressed in the reverse direction. Red dots indicate location of an inverted repeat. Ruler numbers are in kb (1,000 bases). (B) Gepard [23] dotplot showing alignment of the putative transposable region of SecTim467 with the sequence of the *B*. *laterosporus* genome portion where nucleotide homology is present. Numbers show relative nucleotide positions on the genomes. A red dot indicates the location of an inverted repeat.

Common instigators of evolution are transposable elements [87,88]. Phamerator analyses identified a unique region differentiating Jenst from SecTim467. A similar region was also identified in the unrelated phage Sundance. We hypothesize that a putative transposable element found in this region was the cause of this differentiation. The presence of this region in one phage and its absence in another does not appear to be due to a sequencing error. In Jenst, the 1000 bp regions immediately upstream and downstream of the transposon region had a minimum fold coverage of 73 and 71, respectively. In SecTim467, this region had a minimum sequencing fold coverage of 102, with minimum sequencing fold coverages of 125 and 112 of the 1000 bp regions immediately upstream and downstream of the transposon region, respectively. While Sundance also contained genes from this transposable element, they are found in reverse order (Fig 8A). This could either be an instance of segmental flipping due to internal recombination, or the genome of Sundance may be reversed in order. However, it is difficult to determine which of these cases occurred, as, aside from the transposon region, there are only two other small regions of nucleotide homology between these three genomes that did not facilitate sure indication of genome orientation.

BLASTP [39–41] hits from this region of SecTim467 also revealed homology to proteins from *B*. *laterosporus* (gp67 [ALA07413.1] has a homolog to a predicted uncharacterized protein that is 73% similar [WP_003345619.1], gp69 [ALA07415.1] has a homolog to a predicted DNA-binding protein that is 89% similar [WP_003345620.1], gp70 [ALA07575.1] has a homolog that is 96% similar to *B*. *laterosporus* Spo0E [WP_003345621.1], and gp71 [ALA07416.1] has a homolog that is 81% similar to a putative transcriptional regulator [WP_003345622.1]). Homology between SecTim467 and *B*. *laterosporus* was also seen by nucleotide dotplot (Fig 8B). While no conserved domain for a transposase gene was found within this region, a protein homologous to IstB, which may function as a transposase [89,90], was identified elsewhere in the genomes of Jenst (gp146 [YP_009199207.1]) and SecTim467 (gp151 [ALA07476.1]). Pallindrome [60] showed the presence of inverted repeats flanking either side of the putative transposon region in all three phages, which may be involved in transposase insertion and excision from the genomes (S1 Fig) [91]. The similarity in the locations of these inverted repeats in both Jenst and SecTim467, as marked by the red dots in Fig 8A, can be accounted for due to their overall genomic similarities, excluding the putative transposon region itself. The putative transposon region was flanked by conserved inverted repeats designated as bps 65,982–65,997…66,014–66,029 to 66,273–66,282…66,319–66,328 in Jenst and bps 65,982–65,997…66,014–66,029 to 70,414–70,423…70,460–70,469 in SecTim467 (marked as red dots in Fig 8A and also shown in S1 Fig). Only one of the inverted repeats (presented in S1 Fig) is shared between Sundance and the other two phages, and the second is found in the middle of the transposon region in Sundance, making it difficult to define the ends of the putative transposon in this phage. While it appears that this region, or fragments thereof, is also present in the host based on BLAST hits of the transposon region, it is difficult to determine whether SecTim467 or *B*. *laterosporus* was the first to acquire the transposon region since this region shows a different GC content when compared to either chromosome. GC content between SecTim467’s putative transposon and the region of greatest homology in *B*. *laterosporus* LMG 15441 is 34.75% versus 33.42%, respectively. However, the SecTim467 chromosome is 42.71% GC and the host whole genome contains 41.09% GC, indicating that this transposon region was more recently acquired by both SecTim467 and its host in comparison to the rest of their genomes [92].

Further evidence for the excision of this region from Jenst is derived from the remnant of a single gene (Jenst gp67 contrasted to SecTim467 gp71). Although a significant portion of the gene coding for gp67 in Jenst appears to be missing, the DNA binding domain was preserved and may be functional. HHPred [53] protein-folding predictions accessed from the RCSB Protein Data Bank showed that only slight differences existed in the predicted protein folding structure arising from this conserved region. Jenst gp67 matched to the crystal structure of phage P22’s c2 repressor [2R1J [85]] with a probability of 99.6% and an E-value of 4.9e-15 and SecTim467 gp71 matched to *Bacillus thuringiensis* PlcR/PapR7 complex [3U3W [93]] with a probability of 100% and an E-value of 2.8e-29.

It is difficult to determine whether Jenst or SecTim467 was the progenitor as the GC content of these two phages flanked by the inverted repeats is very similar [92], with the average GC content for the transposon region being 36.89% in Jenst, and 37.19% in SecTim467, compared to 42.89% and 42.71% for the whole phage genomes, respectively.

## Conclusions

Herein we have reported the isolation and characterization of five novel *Brevibacillus* phages and their comparison to the five previously reported *Brevibacillus* phages. The detailed analysis of these ten phages lays the foundation for future work concerning the *Brevibacillus* phages, as well as related phages, and aids in understanding and affirming the dynamics of phage-host co-evolution. The genomic comparison and clustering of these phages, along with the identification of putative transcriptional units and proteins, may aid in understanding these phages’ lifecycles. In addition, the identification of host-related proteins and a putative transposon region found in both the phage and host underscore intricate relationships between phages and the evolution of their host.

Whole genome nucleotide dotplot and ANI analysis of all ten *Brevibacillus* phages identified two phage clusters and two singleton phages—a proportional one cluster, one singleton increase from the previously reported one cluster and one singleton [16]. Powder and Osiris were grouped into the previously defined Jimmer-like cluster, a novel cluster containing Jenst and SecTim467 was formed, Emery remained a singleton, and the novel singleton phage, Sundance, was discovered (see Fig 2). These clusters were supported by whole proteome analysis (Table 4) and phylogenetic analysis of a single protein (the large terminase, Fig 4). The classification of these clusters elucidates interesting characteristics where cluster members are very similar to one another while there is a marked dissimilarity between clusters as evidenced by whole genome maps (see Fig 3). What further contrasts these inter-cluster relationships is that many gene products appear to be more similar to phages from distantly related hosts rather than to a *Brevibacillus* phage from another cluster. This trend has been previously reported for the *Enterobacteriaceae* phages and may be due to a small sampling size compared to the large reservoir of phages and their hosts, or due to the ability of phages to acquire new hosts [3]. For example, it is possible that phages may provide an advantage within a distinct ecological niche depending on their genomic content, but as all known *Brevibacillus* phages have been isolated from samples gathered in Utah (and only ten have been fully sequenced thus far), this will remain unclear until more phages are isolated from more locations. However, it is likely that distinct evolutionary lines will still be seen between many clusters even as a large phage reservoir is sequenced, since different clusters may contain phages of very different lifestyles (for example, myoviruses versus siphoviruses). The addition of these five *Brevibacillus* phages, representing two new phage ‘cluster types’, thus provides greater opportunity for use in phage therapy as therapies are more likely effective when they contain phages from different clusters, providing diversity in the different strains they are able to infect. Such therapies may prove to be useful in the treatment of European Foulbrood or other infections where BL is a documented secondary invader [6].

Genomic analysis of these phages revealed a shared promoter-like motif in the Jenst-like cluster, which was located upstream of ORFs whose encoded proteins may predominately function in DNA metabolism (see Fig 5). Thus, these phages may be regulating DNA metabolism proteins for coordinated expression [68,69]. Interestingly, this motif was also found in Basilisk, an unrelated *Bacillus* phage. Further studies are necessary to characterize this motif, including analyzing the expression of the genes downstream of this motif to see if they are coordinated and measuring the infection time to expression of these genes [69,94].

An analysis of the proteomic content of the *Brevibacillus* phages revealed that, in addition to structural proteins, several proteins that may be important for host virulence or the evolution of transcriptional regulation were present. Putative virulence factors identified in the analysis of *Brevibacillus* phage genomes (Table 5) included genes that may affect host pili expression which have been implicated in phage adsorption [95,96] and secretion mechanisms shown to coordinate bacterial colonization of certain cell types [97]. These putative genes support a role for *Brevibacillus* phages in the evolution of pathogenic strains and a potential “Trojan Horse” host entry mechanism employed by the phages that should be studied further. The presence of these genes highlights the need for further phage isolation and analysis in order to determine the contribution of *Brevibacillus* phages to the pathogenicity of their hosts.

Along with virulence factors, several copies of a helix-turn-helix transcriptional regulator were identified in phages within the Jimmer-like cluster. The independent evolution of paralogous genes was analyzed by comparing these proteins (Jimmer1 gp53 and gp57, Jimmer2 gp53 and gp57, Osiris gp55 and gp59, and Powder gp55 and gp59). This helix-turn-helix transcriptional regulator has homology to the classic P22 c2 repressor and may thus be involved in the regulation of the lysogenic state [83,84]. These proteins were placed into two groups of closer homologs, and the amino acid substitutions identified in these different groups were predicted to confer differential nucleotide binding (Fig 6, Table 6). Since gene duplication and subsequent differentiation and selection appears to be a robust method for the evolution of new genes [79–81], these repeated genes offer a window into the evolution of phages and proteins in general. The study of these genes may also provide key insights into the lifecycles of these phages.

Finally, the presence of a putative transposon in both SecTim467 and Sundance, but which was either removed from SecTim467 to create Jenst or added to Jenst to create SecTim467, provides a novel mechanism of driving gene exchange and evolution between these phages and their host, expanding the base of understanding of how these entities have evolved over time (see Fig 8). While the insertion or deletion of a transposable unit is a common evolutionary mechanism [87,88], to our knowledge, this is the first recorded instance of the deletion of a transposon unit being the sole instigator of an evolutionary divergence between two phages. Additionally, by locating a region in the *Brevibacillus* bacterial chromosome that is homologous to this transposable region, the phages’ ability to access the bacterial gene pool is underscored. Future investigation of the functions of the hypothetical proteins in the putative transposable region could prove fortuitous in understanding whether or not this region contributes to host pathogenicity and function.

## Supporting Information

S1 Fig Inverted repeats flank either side of the transposable regions found in Jenst, SecTim467, and Sundance. Pallindrome [60] was used to verify the presence of inverted repeats surrounding the transposable regions of Jenst, SecTim467, and Sundance. Each column contains the inverted repeats present in and around the transposon region for each of the phages and each row contains the predicted hairpin binding of the nucleotide sequence.
(TIF)

S1 Table Analysis of conserved proteins encoded by the ten *Brevibacillus* phages. (XLSX)
