# Supplementary figures and images for: Characterization of Five Novel Brevibacillus Bacteriophages and Genomic Comparison of Brevibacillus Phages
Source: PLoS One. 2016 Jun 15;11(6):e0156838. doi: 10.1371/journal.pone.0156838 (PMC4909266; doi:10.1371/journal.pone.0156838)

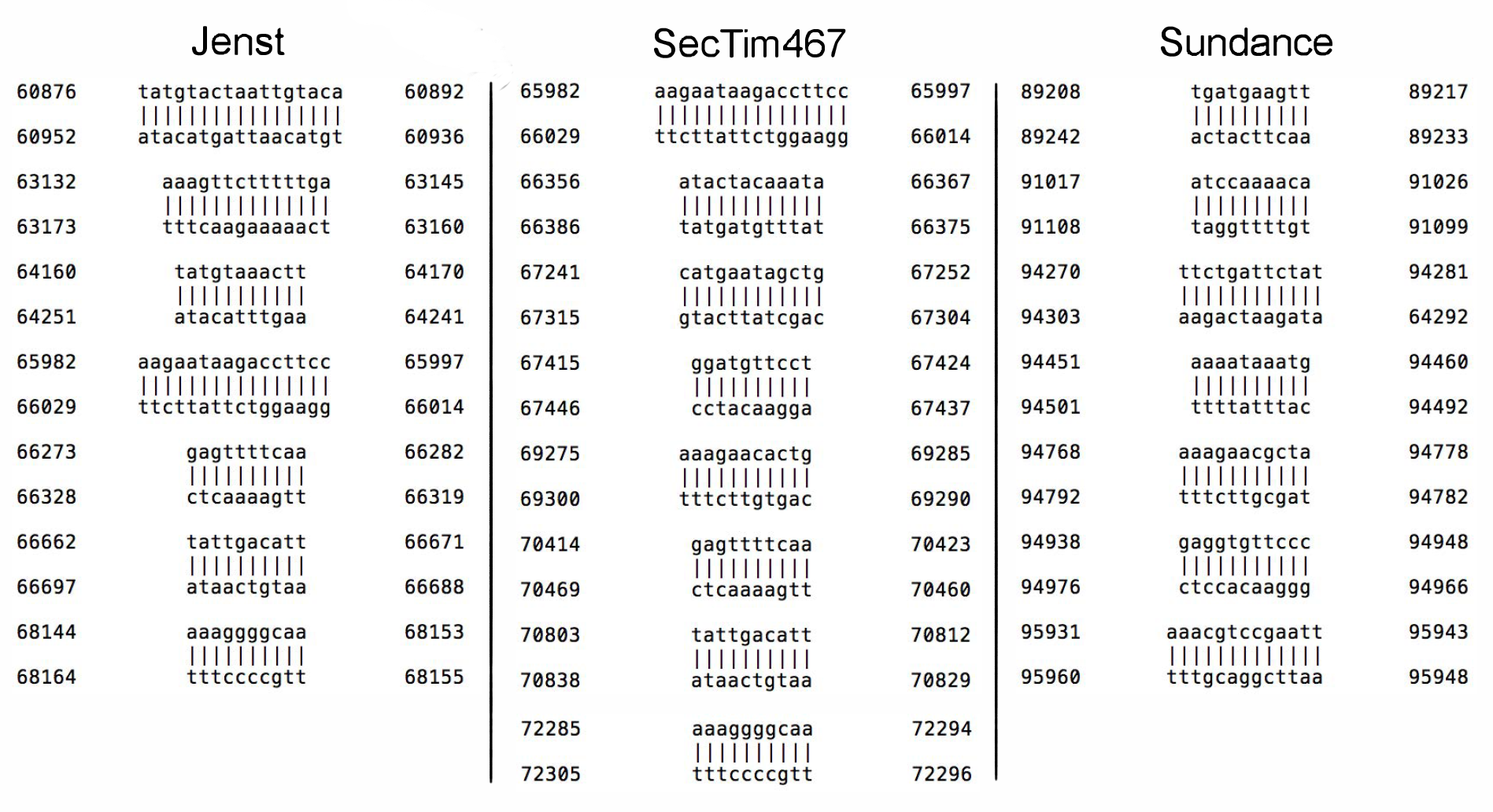

Supplement: S1 Fig — Pallindrome [60] was used to verify the presence of inverted repeats surrounding the transposable regions of Jenst, SecTim467, and Sundance. Each column contains the inverted repeats present in and around the transposon region for each of the phages and each row contains the predicted hairpin binding of the nucleotide sequence. (TIF) [file pone.0156838.s001.tif]
